# RSK/GSK3–mediated phosphorylation of FilGAP regulates chemotactic cancer invasion

**DOI:** 10.1093/pnasnexus/pgae071

**Published:** 2024-02-09

**Authors:** Koji Tsutsumi, Yasutaka Ohta

**Affiliations:** Division of Cell Biology, Department of Biosciences, School of Science, Kitasato University, Minami-ku, Kanagawa 252-0373, Japan; Division of Cell Biology, Department of Biosciences, School of Science, Kitasato University, Minami-ku, Kanagawa 252-0373, Japan

**Keywords:** chemotaxis, EGF, p90RSK, GSK3, cell migration

## Abstract

Cell migration plays a crucial role in various biological processes, such as gastrulation, immune response, and cancer metastasis. In response to chemoattractant-like growth factors, cells form protrusions and migrate toward the source of the signal. Rho family small GTPase Rac is a key regulator of cell migration by stimulating actin polymerization to generate lamellipodia, flat membrane protrusions at the leading edge of migrating cells. FilGAP (ARHGAP24), a Rac-specific GTPase-activating protein (GAP), suppresses lamellipodia formation, and controls tumor cell migration. In this study, we found that FilGAP is phosphorylated downstream of epidermal growth factor (EGF) signaling. Upon EGF stimulation, FilGAP is phosphorylated at Ser625 by p90 ribosomal S6 kinase (RSK) and then at Ser621 by glycogen synthase kinase 3 (GSK3). Phosphorylation of FilGAP induces its dissociation from actin filaments. We identified a novel actin-localization domain of FilGAP that is essential for stabilizing cell adhesion. Additionally, we found that phosphorylation of FilGAP inhibits its lamellipodia suppression activity. Finally, we showed the expression of nonphosphorylatable FilGAP mutant, but not wild-type FilGAP, reduced cell migration speed and persistence toward the EGF gradient. Taken together, our results suggest that phosphorylation of FilGAP downstream of EGF-signaling plays a critical role in regulating chemotactic tumor cell migration by controlling cell–matrix adhesion and protrusion formation.

**Significance Statement**

Cancer cells spread by invading the extracellular matrix and metastasizing to other organs. Rac is required for cancer cells to generate protrusions in the direction in which they receive chemoattractant. Although the activation pathway of Rac in chemotaxis has been extensively studied, the inactivation pathway has been less characterized. Here we have defined FilGAP, a negative regulator of Rac1, is phosphorylated by p90 ribosomal S6 kinase (RSK) and glycogen synthase kinase 3 (GSK3) downstream epidermal growth factor (EGF), thereby promoting cancer cell chemotactic invasion. FilGAP regulates cancer cell chemotaxis by modulating two distinct functions, cell protrusion, and cell adhesion, through distinct spatial localizations regulated by phosphorylation. These data provide insights into how Rac GTPase-activating protein is regulated and behaves during cancer cell chemotaxis.

## Introduction

Cell migration is a fundamental biological process in various biological events, including early embryonic development such as gastrulation, epithelial wound closure, and immune response (1). Cells can sense their surrounding environment, such as small molecules (e.g. cytokine or growth factors), extracellular matrix, and alter their morphology to determine the direction of movement. Chemotaxis is a directional cell migration toward the direction of a chemical gradient (2). Tumor cells show chemotactic properties against chemokines and growth factors and it contributes to the tumor cell dissemination (3).

Epidermal growth factor (EGF) is one of the most studied growth factors involved in cancer cell chemotaxis. Upon binding to EGF receptors, cells rapidly reorganize the actin cytoskeleton and cell adhesions, acquire polarized shape, and begin to migrate. Rho family small GTPases (Rho GTPases), including Rac, Rho, and Cdc42, are key regulators of actin cytoskeleton reorganization (4, 5). There are many reports of Rho GTPases activation in EGF signaling. For example, EGF receptor directly phosphorylates Vav2, a Guanine nucleotide exchange factor (GEF) for Rac, and this phosphorylation increases its GEF activity (6). Although the activation of GEFs in chemotaxis has been well studied, how the GTPase-activating protein (GAPs) behave in chemotaxis is still not well understood.

One of the major signaling pathways downstream of EGF is mitogen-activated protein (MAP) kinase cascade (7). Raf, mitogen-activated protein kinase kinase (MEK), and extracellular signal-regulated kinase (ERK) are sequentially phosphorylated and activated ERK phosphorylates cytosolic targets and nuclear transcription factors (8). p90 ribosomal S6 kinases (RSKs) 1 and 2, which are cytosolic targets of ERK, are serine/threonine kinases that regulate various cellular processes, including cell growth, proliferation, and motility, through target phosphorylation (9, 10). RSK regulates cell adhesion and actin cytoskeleton through direct phosphorylation of its substrates in cancer cells (11–14). RSK phosphorylates filamin A at Ser2152 and inhibits α5β1 integrin-dependent adhesions (15, 16). RSK2 phosphorylates leukemia-associated Rho GEF (LARG/ARHGEF12), a GEF for Rho, which is required for cell migration and invasion in glioblastoma cells (17). However, the role of RSK in cancer cell chemotaxis is not well understood.

Glycogen synthase kinase 3 (GSK3) α and β are multifunctional serine/threonine kinases that are ubiquitously expressed in a variety of tissues (18). GSK3 influences cell migration through the regulation of cytoskeletal dynamics and cell–matrix interaction (19, 20). GSK3 is thought to function upstream of RhoGTPases in cell motility and polarity formation (21), but the direct regulation of GEFs and GAPs by GSK3 is not well understood.

FilGAP (ARHGAP24) is a Rac-specific GAP, isolated as a filamin A binding protein (22, 23). FilGAP regulates epithelial cell adhesion and tubulogenesis (24, 25), as well as cancer cell morphology and invasion (26–28). FilGAP is phosphorylated at several serine/threonine residues, including Ser402, and this phosphorylation regulates GAP activity and cytoskeletal localization of FilGAP (29). While FilGAP regulates EGF-induced lamellipodia formation and cell spreading on extracellular matrices (22, 30), how FilGAP is regulated in these processes remains poorly understood.

Here we demonstrate that FilGAP is phosphorylated by RSK and GSK3 downstream of EGF signaling. Phosphorylation leads to the release of FilGAP from actin cytoskeleton. FilGAP functions to suppress lamellipodia formation and stabilize cell adhesion to extracellular matrices; however, phosphorylation attenuates these processes. Finally, we have revealed that phosphorylation of FilGAP is required for efficient cancer cell chemotaxis toward the EGF gradient.

## Results

### FilGAP is phosphorylated downstream EGF signaling

**Fig. 1.**
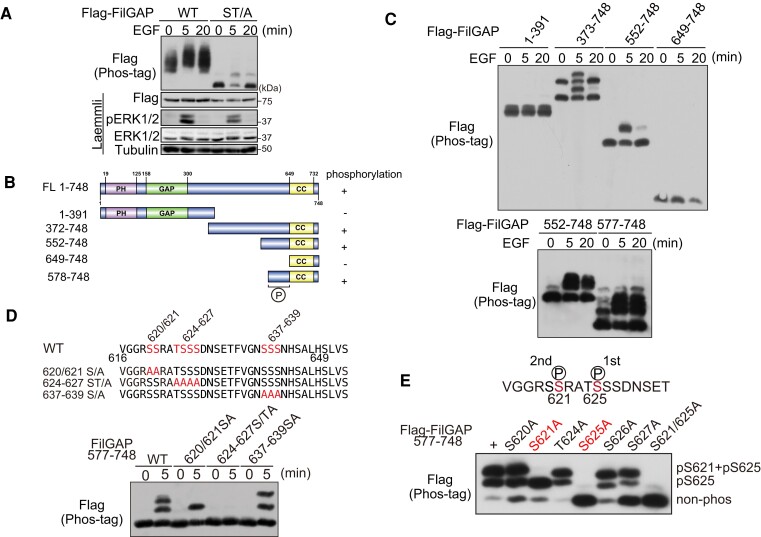
FilGAP is sequentially phosphorylated at Ser625 and Ser621 downstream of EGF. A) HEK293T cells transfected with Flag-FilGAP (WT or S/TA) were treated with 50 ng/mL EGF for the indicated time. Phosphorylation was analyzed by Phos-tag SDS–PAGE. “Laemmli” is the results of SDS–PAGE without Phos-tag, showing that no band-shift was observed and that the same amounts of protein were applied. B) Schematic representation of FilGAP deletion constructs. C) HEK293T cells transfected with Flag-FilGAP deletion constructs were treated with EGF. Phosphorylation was analyzed by phos-tag SDS–PAGE. D) There are several serine or threonine clusters in the C-terminal region of FilGAP. Mutant FilGAP constructs in which each serine/threonine cluster was substituted to alanine (top panel) were expressed in HKE293 cells and treated with EGF for 30 min. Phosphorylation was analyzed by phos-tag SDS–PAGE. E) FilGAP single alanine mutants were expressed in HEK293T cells and treated with EGF for 30 min. Phosphorylation was analyzed by phos-tag SDS–PAGE.

FilGAP can be phosphorylated by various stimuli, including lysophosphatidic acid (LPA) or serum stimulation, as we previously reported (22, 26). To investigate whether other growth factors can also phosphorylate FilGAP, we used Phos-tag analysis (31). Flag-FilGAP was transfected in HEK293T cells, and the cells were stimulated with EGF. Flag-FilGAP was detected at a slightly slower position after EGF treatment, suggesting that FilGAP was phosphorylated downstream of EGF signaling (Fig. 1A). Interestingly, FilGAP-ST/A mutant, in which six phosphorylation sites (Ser391, Ser402, Ser413, Ser415, Ser437, and Thr452) of FilGAP by multiple kinases including ROCK were replaced with alanine, also showed an upper-shifted band after EGF treatment, suggesting that a different amino acid residue was phosphorylated other than the mutated sites. Furthermore, we generated various deletion mutants and examined whether they were phosphorylated by EGF (Fig. 1B). 1–391 aa and 649–748 aa were not phosphorylated, while 577–748 was phosphorylated, suggesting that there is a phosphorylation site at 577–748 in response to EGF, although 392–577 may still contain phosphorylation sites (Fig. 1C). We found several Ser/Thr clusters in this region (Fig. 1D) and generated Ala-substituted mutants (S620/621A, S/T624-627A, S637-639A) to examine which ones were phosphorylated. The results showed that phosphorylation sites were located in 620–621aa and 624–627aa. We then generated single alanine substituted mutants (S620A, S621A, T624A, S625A, S626A, and S627A) and examined their phosphorylation (Fig. 1E). S621A showed one upper-shifted band and S625A did not show any shifted band, and other mutants showed two upper-shifted bands. These data suggest that Ser621 and Ser625 may be phosphorylated downstream of EGF, and phosphorylation of Ser625 may be required for Ser621 to be phosphorylated initially.

### FilGAP is sequentially phosphorylated by RSK and GSK3

**Fig. 2.**
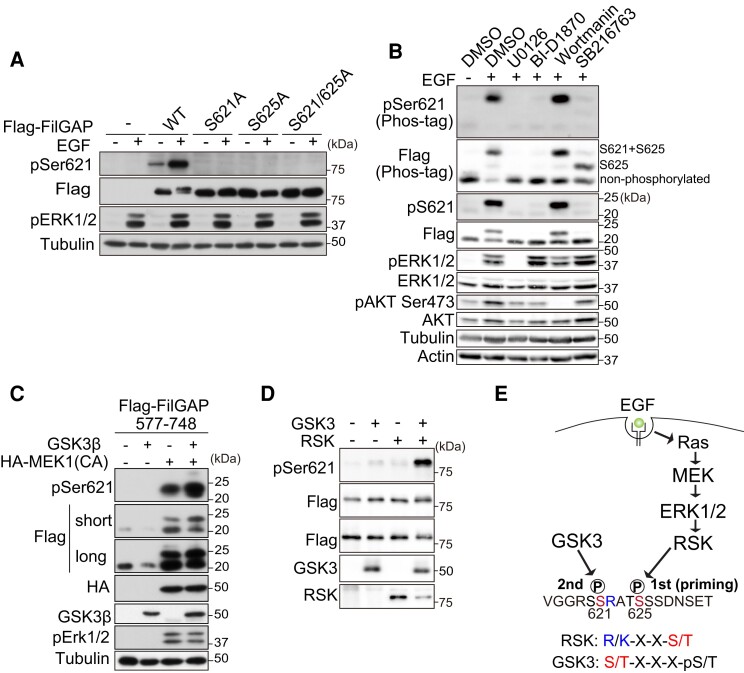
FilGAP is sequentially phosphorylated by RSK and GSK3. A) A7 melanoma cells transfected with Flag-FilGAP (WT, S621A, S625A, and S621/625A) were treated with EGF for 30 min. Cell lysates were analyzed by western blotting by indicated antibodies. B) Flag-FilGAP 577–748 was expressed in HEK293T cells and treated by Dimethyl sulfoxide (DMSO), U0126, BI-D1870, Wortmanin, or SB216763 for 30 min before EGF addition. Phosphorylation was analyzed by phos-tag SDS–PAGE. C) HEK293T cells transfected to indicated plasmids were analyzed by western blotting analysis. D) Purified Flag-FilGAP was incubated with purified Flag-GSK3β or RSK immunoprecipitate in presence of ATP for 30 min. Phosphorylation of FilGAP was analyzed by western blotting. E) Schematic diagram of regulatory mechanism of FilGAP phosphorylation downstream of EGF and phosphorylation consensus sequence of RSK and GSK3. Phosphorylation sites were shown in red and the basic residue, which is required for RSK consensus sequence motif, was shown in blue.

Ser625 has been reported to be phosphorylated by phosphoproteomics, but Ser621 has not yet been reported in any papers or mass spectrometry analyses (32). To detect Ser621 phosphorylation, we generated a phospho-specific antibody against Ser621 (anti-pSer621) and tested its specificity (Fig. 2A). After EGF treatment, the anti-pSer621 antibodies strongly reacted against FilGAP wild-type (WT) but not with S621A or S625A mutant, indicating that Ser621 is phosphorylated in the cells after EGF treatment. To explore the kinases responsible for phosphorylation of Ser621 and Ser625, HEK293T cells transfected with FilGAP 577–748, whose phosphorylation of Ser621 and Ser625 can be easily distinguished by Phos-tag SDS polyacryl amide gel electrophoresis (SDS–PAGE), were treated with EGF in the presence of several kinase inhibitors (U0126: MEK inhibitor, BI-D1870: RSK inhibitor, wortmannin: phosphatidylinositol-3 kinase [PI3K] inhibitor, SB216763: GSK3 inhibitor) (Fig. 2B). MEK and RSK inhibitor completely suppressed both upper and lower upshifted bands of FilGAP after EGF treatment. Interestingly, GSK3 inhibitors suppressed only the upper upshifted band of FilGAP as well as S621A (Fig. 1E), while the lower upshifted remained, suggesting that GSK3 may phosphorylate Ser621. We then transfected HEK293T cells with Flag-FilGAP, with or without constitutively activated (CA) MEK1 and GSK3β (Fig. 2C). GSK3 overexpression did not promote FilGAP-Ser621 phosphorylation. However, CA-MEK1 overexpression promotes Ser621 phosphorylation, and phosphorylation was further increased when both CA-MEK1 and GSK3 were transfected. To clarify whether RSK and GSK3 directly phosphorylate FilGAP, we performed in vitro kinase assay. Flag-FilGAP was expressed in HEK293T cells and purified by anti-Flag sepharose. Purified Flag-FilGAP was incubated with purified GSK3 and RSK alone or combination of both in the presence of adenosine triphosphate (ATP) (Fig. 2D). Anti-pSer621 signal was detected only when incubated with both RSK and GSK3, indicating that both RSK and GSK3 are required for the phosphorylation of Ser621of FilGAP. It is well known that GSK3 required priming phosphorylation at the +4 position of its phosphorylation site. Furthermore, Ser625 has Arg residue at −2 position, matching the RSK phosphorylation consensus sequence (R-X-X-S). Based on this evidence, we concluded that Ser625 is phosphorylated by RSK, and then Ser621 is phosphorylated by GSK3 (Fig. 2E).

### Phosphorylation changed the cytoskeletal localization of FilGAP

**Fig. 3.**
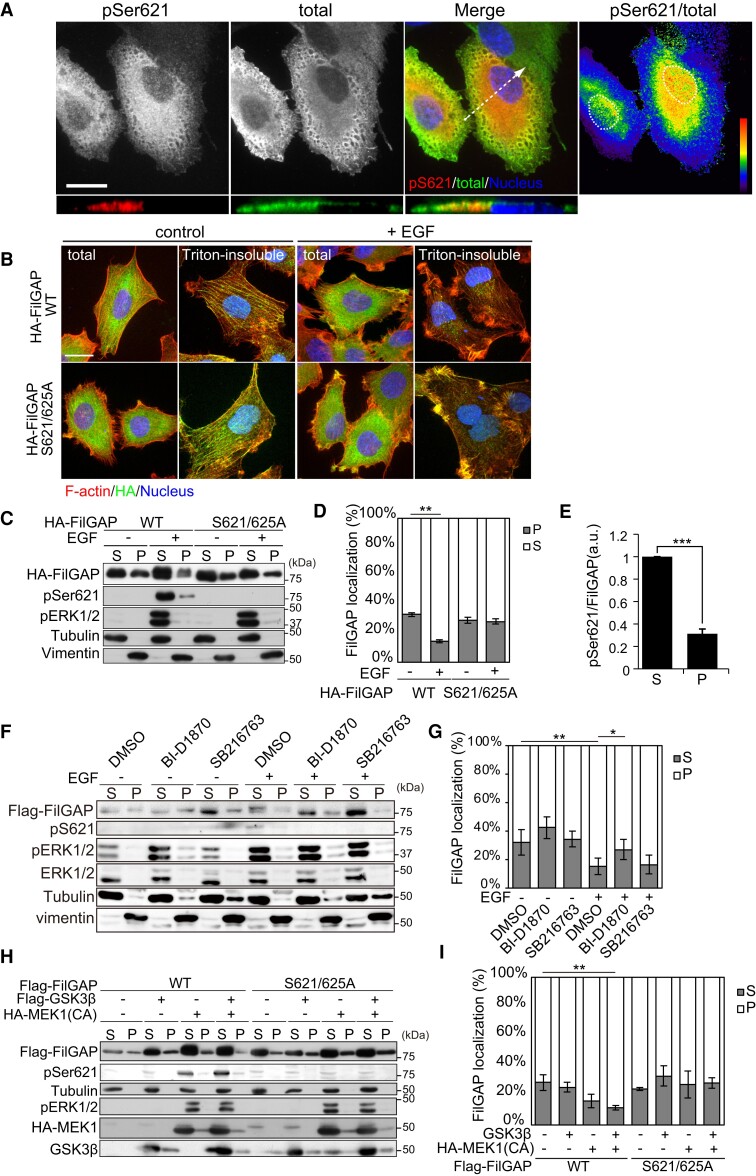
Phosphorylation of FilGAP by RSK and GSK3 regulates its cellular localization. A) A7 cells were transduced with HA-FilGAP and incubated with EGF for 30 min, and then fixed and stained with anti-pSer621 and anti-HA antibodies. The ratio image was generated by dividing pSer621signal by HA signal. ZY slices at arrow were shown below. The dotted circle indicates the position of nucleus. Scale bar: 20 μm. B) HA-FilGAP (WT or S621/625A) transduced A7 cells were incubated in the presence or absence of EGF for 30 min. Cells were fixed after treatment with (Triton-insoluble) or without (total) 0.5% Triton X-100 and stained with anti-HA antibodies for FilGAP and Alexa568 conjugated phalloidin for F-actin. Scale bar: 20 μm. C) HA-FilGAP (WT or S621/625A) transduced A7 cells were incubated in the presence or absence of EGF for 30 min. The cells were lysed, and Triton X-100 solubilized (S) and insoluble cytoskeletal fractions (P) were prepared. Samples of each fraction were analyzed by immunoblotting with indicated antibodies. D) The proportion of HA-FilGAP localization was shown. *n* = 3, *** *P*< 0.001 (Student's t test). E) The relative amounts of HA-FilGAP phosphorylated at Ser621 compared to total HA-FilGAP were shown. *n* = 3, *** *P*< 0.001 (Student's t test). F) Flag-FilGAP transduced A7 cells were treated by DMSO, BI-D1870, or SB216763 for 60 min prior to EGF addition. The cells were lysed, and Triton X-100-solubilized (S) and insoluble cytoskeletal fractions (P) were prepared. Samples of each fraction were analyzed by immunoblotting with indicated antibodies. G) The proportion of Flag-FilGAP localization was shown. *n* = 5, *P< 0.05, **P< 0.01 (Student's t test). H) HEK293T cells transfected to indicated plasmids were subjected to cell fractionation assay. I) The proportion of Flag-FilGAP localization was shown. *n* = 3, *P< 0.05 (Student's t test).

Protein phosphorylation adds the acidic property to the target protein and often leads to a change in its subcellular localization. To examine whether the phosphorylation of FilGAP affects its subcellular localization, human melanoma A7 cells were transfected with Flag-FilGAP, and stained with anti-pSer621 and anti-Flag antibody. Before EGF treatment, anti-pSer621 signal was mostly undetectable (Fig. S1A). However, after EGF treatment, anti-pSer621 signal was greatly increased, indicating that phosphorylation of Ser621 occurs in response to EGF. Anti-pSer621 signal appears to be reduced at the cell periphery (Fig. 3A). This was more evident from ratio image of pSer621 divided by total FilGAP, suggesting that phosphorylation of FilGAP is involved in the regulation of its subcellular localization.

We previously reported that FilGAP localized at actin cytoskeleton, and phosphorylation releases the FilGAP from actin cytoskeleton (29). Short-time treatment with Triton X-100 before fixation removed FilGAP from the cytosol, FilGAP showed clear colocalization with actin filaments (Figs. 3B and S2B). After EGF treatment, the cytoskeletal localization of FilGAP was decreased. To test whether phosphorylation of FilGAP downstream EGF is involved in the actin cytoskeletal localization, we examined the cellular localization of nonphosphorylatable FilGAP S621/625A mutant. S621/625A localized on actin cytoskeleton, even after EGF stimulation. Further, we examined the cytoskeletal localization of FilGAP by biochemical fractionation. The amount of FilGAP WT in Triton X-100 insoluble precipitate was decreased after EGF treatment, while the amount of FilGAP in soluble supernatant was significantly increased (Fig. 3C and D). Moreover, FilGAP phosphorylated at Ser621 was mostly collected in the soluble fraction, and only some in the insoluble precipitate (Fig. 3E). The reduction in the amount of FilGAP in the precipitates by EGF treatment was blocked by RSK inhibition but not by GSK3 inhibition (Fig. 3F and G). Co-expression with constitutive active MEK1 and GSK3 also reduced the amount of FilGAP in the insoluble precipitate (Fig. 3H and I). These data suggest that FilGAP is dissociated from actin cytoskeleton by phosphorylation by RSK and GSK3 downstream EGF.

### Basic amino acids of FilGAP are critical for its actin cytoskeletal localization

**Fig. 4.**
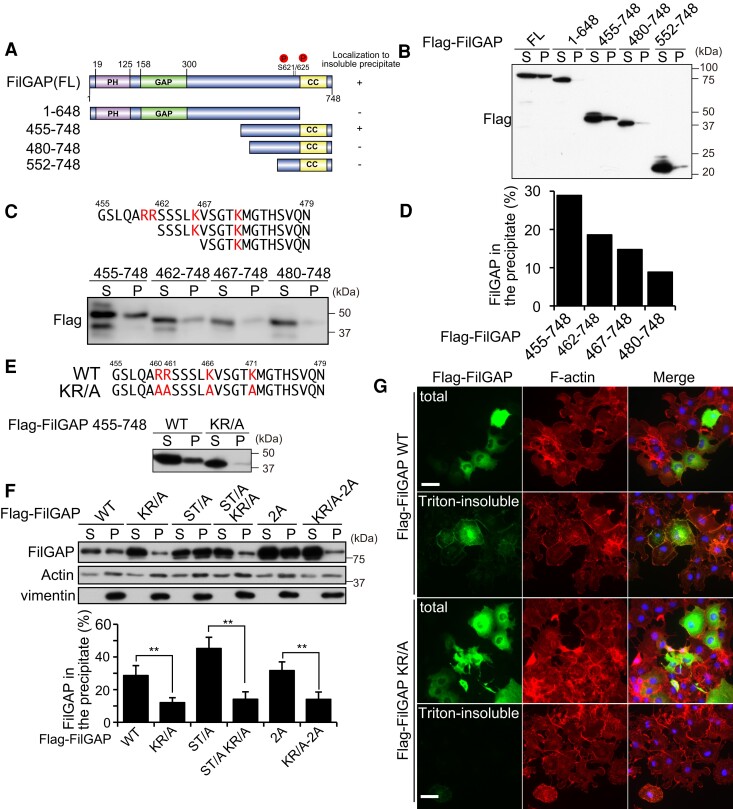
Basic amino acids clusters of FilGAP are necessary for the localization to actin cytoskeleton. A) Schematic representation of the FilGAP deletion mutants and their localization to Triton X-100 insoluble precipitate. B) Triton X-100 insolubility of FilGAP deletion mutants in HEK293T cells was analyzed by western blotting using anti-Flag antibodies. C) Amino acids sequence between 455–479 aa of FilGAP. Basic amino acids are shown in red. Triton X-100 insolubility of FilGAP C-terminal deletion mutants was analyzed by western blotting using anti-Flag antibodies. D) Quantification of the amount of FilGAP in Triton X-100 insoluble precipitate is shown. E) KR/A mutant, in which all basic amino acid residues within 455–479 aa of FilGAP were substituted to alanine, failed to localize to Triton X-100 insoluble precipitates. F) Triton X-100 insolubility of full-length FilGAP mutants was analyzed by western blotting with anti-Flag antibodies. Actin and vimentin were used as a loading control for fractionation. **P < 0.05 (Student's t test). G) COS-7 cells were transfected with Flag-FilGAP (WT, KR/A). After 24 h, cells were fixed after treatment with (Triton-insoluble) or without (total) 0.5% Triton X-100 and stained with anti-Flag antibodies for FilGAP (green) and Alexa568 conjugated phalloidin for F-actin (red). Scale bar: 20 μm.

Next, we examined how FilGAP is associated with the actin cytoskeleton. We expressed FilGAP deletion mutants in HEK293T cells (Fig. 4A) and examined their F-actin localization. C-terminal deletion (1–648 aa) clearly decreases the amount of FilGAP in insoluble precipitates. N-terminal 454 aa deleted FilGAP was still collected in the insoluble precipitate, but N-terminal 480 aa deleted FilGAP was mostly collected in soluble fraction (Fig. 4B). We focused on ****450–480 aa sequence and found that this region includes several basic residues (Fig. 4C). Some actin-binding proteins are associated with the amino-terminal acidic residues of actin via their basic residues (33). As we gradually deleted 455–480 aa of FilGAP from N-terminus, the amount of FilGAP collected in precipitates decreased (Fig. 4C and D). Further, all lysine or arginine residues among 455–480 aa of FilGAP were substituted to alanine (KR/A) and examined the Triton X-100 solubility. Most of FilGAP 455–748 KR/A mutant was collected in soluble fraction, suggesting that basic residues among 455–480 of FilGAP are required for recovery in the insoluble precipitate (Fig. 4E). In addition, solubility in Triton X-100 and F-actin localization were also reduced by the KR/A mutation in full-length FilGAP (Fig. 4F and G). These data indicate that these basic residues seem critical for F-actin localization for FilGAP.

### Phosphorylation of FilGAP regulates cell spreading and lamellipodia formation

**Fig. 5.**
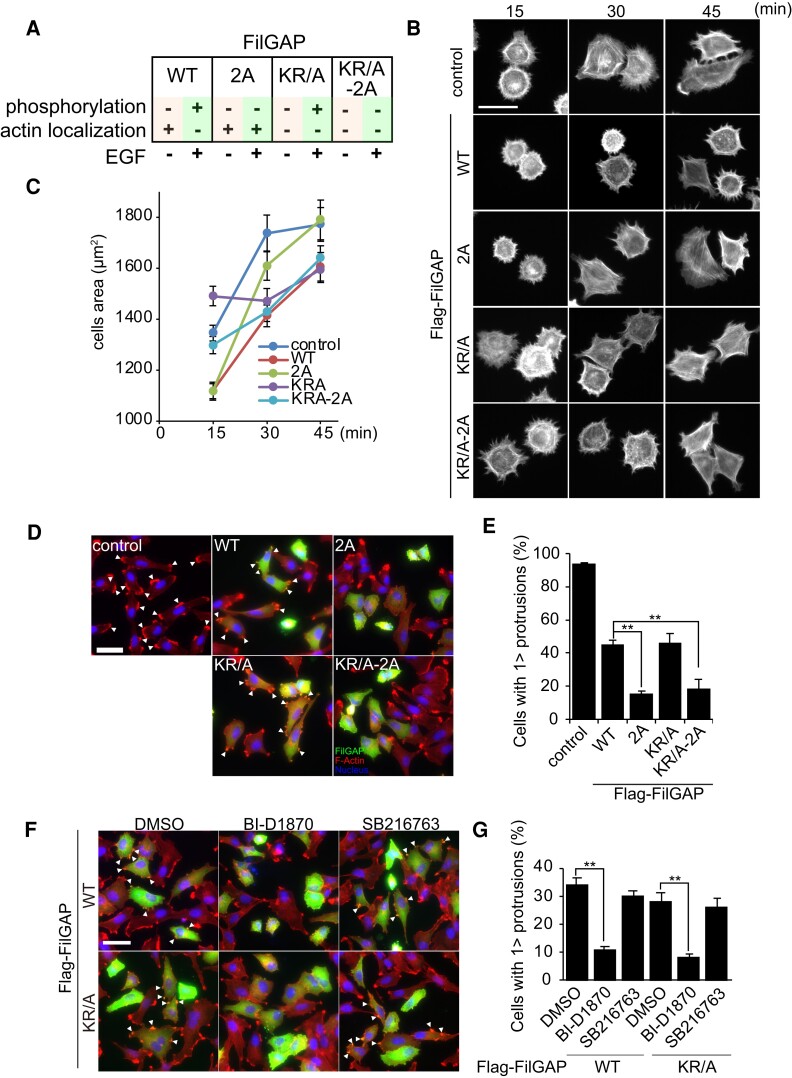
Phosphorylation of FilGAP regulates cell spreading and lamellipodia formation A) Summary of the properties of FilGAP mutants. B) Flag-FilGAP (WT, 2A, KR/A, KR/A-2A) transduced A7 cells were subjected to cell spreading assay on collagen-coated coverslip. Cells were stained with Aelxa 568-conjugated phalloidin after formaldehyde fixation. C) Cell areas at each time point were quantified. D) A7 cells were transfected with Flag-FilGAP and cultured for 20 h in the absence of serum. Twenty-four hours after transfection, cells were treated with EGF for 30 min and then fixed. Asterisk indicates F-actin-rich protrusions induced by EGF. E) Quantification of the cells with actin-rich protrusions. **P < 0.01 (ANOVA and Tukey HDS test). F) A7 cells transfected with Flag-FilGAP were incubated with RSK or GSK3 inhibitors for 2 h, followed by EGF treatment, and then stained for F-actin. G) Quantification of the cells with actin-rich protrusions. **P < 0.01 (ANOVA and Tukey HDS test). Scale bars: 50 μm.

FilGAP is localized to various cellular components including actin cytoskeleton, plasma membrane, and cytosol (29, 34). To investigate how different subcellular localization and phosphorylation affect the function of FilGAP, we generated a set of mutants that combined actin-localization deficient mutations (K/RA) and nonphosphorylatable mutations (S621/625A; 2A) (Fig. 5A). FilGAP WT can associate with actin cytoskeleton, but is phosphorylated downstream EGF and leading to dissociation from actin cytoskeleton. In contrast, FilGAP 2A mutant can associate with actin cytoskeleton even after EGF stimulation. Since KR/A mutation impairs actin cytoskeletal localization of FilGAP, FilGAP K/RA mutants (KR/A and KR/A-2A) can be used to investigate the role of phosphorylation of FilGAP that does not localize to actin cytoskeleton. To investigate the role of phosphorylation and the actin cytoskeletal localization of FilGAP, we used A7 melanoma cells, which spread well on extracellular matrix, respond well to EGF and form lamellipodia (22, 30). We generated A7 cells stably expressing FilGAP (WT, 2A, KR/A, and KR/A-2A) and performed a cell spreading assay on collagen-coated coverslip. In the early stage (15 min) of cell spreading, cell areas were significantly decreased in FilGAP WT, 2A-expressing cells, but not in FilGAP KR/A and KR/A-2A-expressing cells (Fig. 5B and C and Movie S1). However, in the late stage of cell spreading (45 min), cell areas were significantly decreased in FilGAP WT, KR/A, and KR/A-2A-expressing cells but not in FilGAP 2A-expressing cells. Early adhesion involves Rac-dependent lamellipodia formation and cell spreading, while late adhesion involves stress fiber formation and focal adhesion (FA) maturation through RhoA activation (35). Therefore, we hypothesized that phosphorylation and actin cytoskeletal localization of FilGAP may be involved in the formation of lamellipodia and the regulation of FA. We assessed whether phosphorylation of FilGAP at Ser621 and Ser625 was involved in inhibition of lamellipodia formation. A7 cells generated several actin-rich cell protrusions at cell periphery after EGF treatment (Fig. 5D, arrowheads). Expression of FilGAP WT and KR/A suppressed the protrusions formation, but a small number of the cells still generated protrusions (Fig. 5D and E). On the other hand, expression of FilGAP 2A and KR/A-2A-expressing cells strongly suppressed cell protrusions, suggesting that phosphorylation inhibits lamellae suppression activity of FilGAP, regardless of whether FilGAP is associated with the actin cytoskeleton or not. In addition, we examined the effect of RSK and GSK3 inhibition on the lamellipodia inhibitory activity of FilGAP. Inhibition of RSK markedly suppressed the formation of lamellipodia in cells expressing FilGAP WT and KR/A, even though untransfected cells do form lamellipodia (Fig. 5F and G). On the other hand, inhibition of GSK3 did not affect lamellipodia formation in FilGAP-expressing cells. These data suggest that FilGAP, which is not associated with actin cytoskeleton, prevents lamellipodia formation, and phosphorylation inhibits this function.

### Phosphorylation of FilGAP regulates focal adhesion stability

**Fig. 6.**
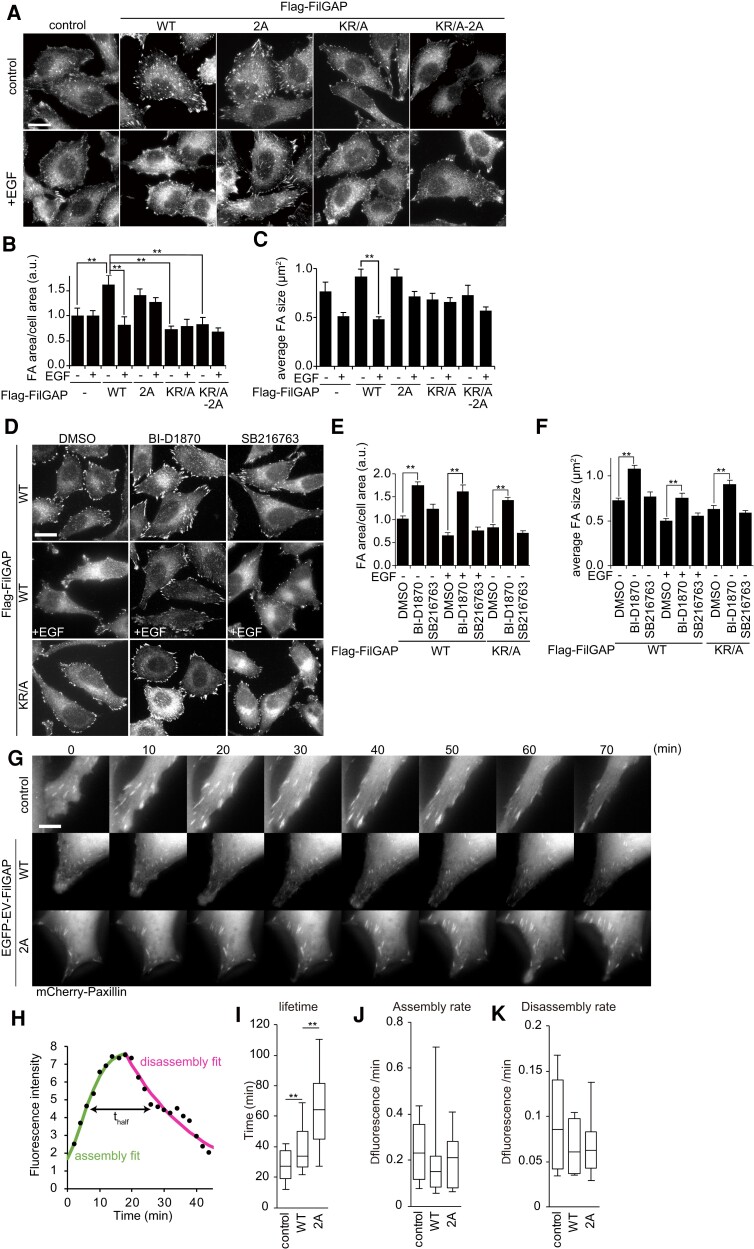
Phosphorylation of FilGAP is involved in regulation of Focal adhesion. A) Flag-FilGAP (WT, 2A, KR/A, KR/A-2A) transduced A7 cells were cultured on collagen-coated coverslip in the absence of serum. Cells were incubated in the presence or absence of EGF for 30 min. Cells were fixed and stained with anti-Paxillin antibodies to visualize focal adhesions. Scale bar is 10 μm. B and C) The area of FA per cell and the size of individual focal adhesions (FAs) were calculated. *n* = 11–13 cells. **P < 0.01 (ANOVA and Tukey HDS test). D) Flag-FilGAP transduced A7 cells were incubated with RSK or GSK3 inhibitors for 2 h, followed by EGF treatment, and then stained for anti-Paxillin. Scale bar: 20 μm. E–F) The area of FA per cell and the size of individual FAs were calculated. *n* = 16–26 cells. **P < 0.01 (ANOVA and Tukey HDS test). G) mCherry-Paxillin transduced A7 cells were transfected with EGFP-EV-FilGAP (WT or 2A) and cultured O/N on collagen-coated glass bottom dish in medium containing the reduced serum (1%). Cells were treated with EGF and observed at 2 min intervals for 6 h. Montage images every 10 min were shown. Scale bar: 10 μm. H) Representative graph of mCherry-Paxillin lifetime curve fitting. I–K) Quantification of mCherry-Paxillin lifetime (I), assembly rate (J), disassembly rate (K). *n* = 20. **P < 0.01 (ANOVA and Tukey HDS test).

Next, we analyzed whether phosphorylation of FilGAP is also involved in regulation of FA. A7 cells stably expressing FilGAP WT, 2A, KR/A, and KR/A-2A were cultured on collagen-coated coverslip and stained with anti-Paxillin antibody to visualize FA (Fig. 6A–C). Expression of FilGAP WT and 2A but not KR/A and KR/A-2A increased the area and the size of FA. Further, after EGF stimulation, the FA areas were reduced in FilGAP WT-expressing cells, but not in FilGAP 2A. Given that FilGAP is released from the actin cytoskeleton by phosphorylation, this suggested that FilGAP associated with cytoskeleton stabilizes FA. We then examined the effects of RSK and GSK3 inhibition on the regulation of FA by FilGAP. RSK inhibition significantly increased the area of FA (Fig. 6D–F). This effect was also observed in KR/A mutant, suggesting that RSK also regulates FA through other pathways than FilGAP. Next, to analyze FA dynamics, A7 cells stably expressing mCherry-Paxillin were generated, and Enhanced green protein (EGFP)-EV-FilGAP, in which a flexible EV linker was inserted between EGFP and FilGAP, was expressed in these cells (Movie S2, Figs. 6G and S3). The fluorescence intensity of mCherry-paxillin was measured over time and the lifetime, assembly rate, and disassembly rate of FA were determined (36) (Fig. 6H–K). Although the assembly rate and disassembly rate were not significantly different, lifetime was slightly increased in WT, with a marked increase observed in 2A. These data suggest that FilGAP on the actin cytoskeleton function to maintain FA, but when they are phosphorylated and released from the actin cytoskeleton, FA is disassembled.

### Phosphorylation of FilGAP downstream EGF regulates chemotactic cancer invasion

**Fig. 7.**
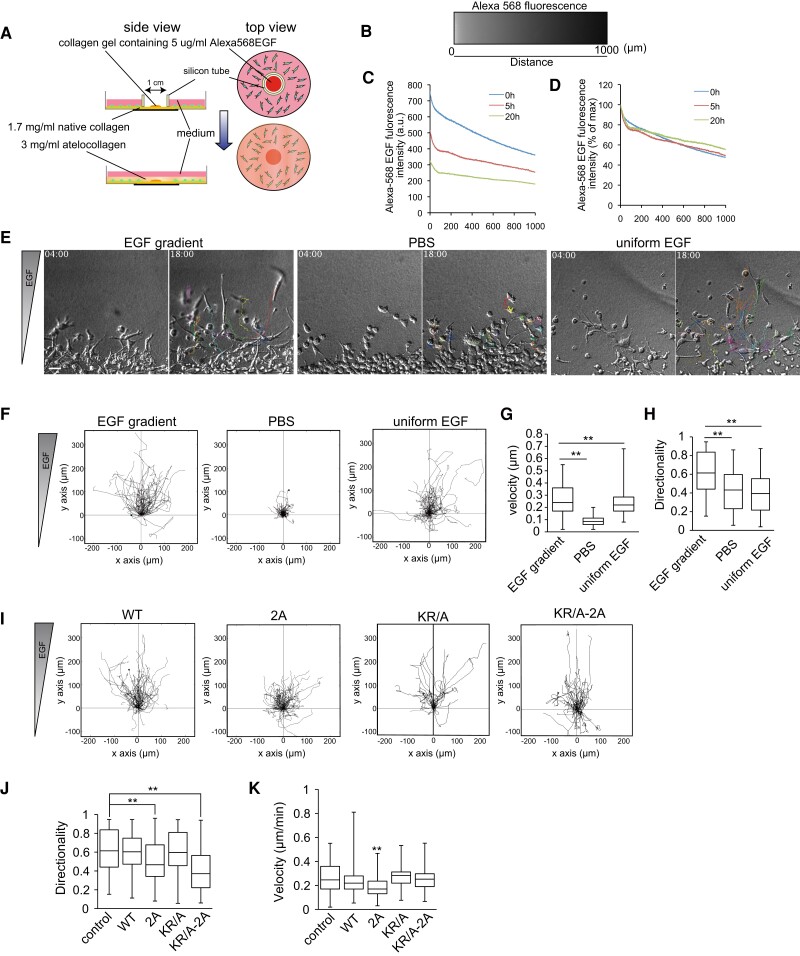
FilGAP regulates chemotactic invasion. A) Schematic representation of three-dimensional EGF chemotaxis setup. B) Alexa568-EGF slope at time 0. C and D) Evolution over time of the intensity (C) and slope (D) of the Alexa568-EGF gradient. E) Differential interference images of the trajectory of A7 cell between 4- and 18-h of chemotaxis invasion assay. Scale bars: 50 μm (F) Summary of trajectory in (E). G and H) The velocities and directionalities of migrating cells were calculated, and the data were expressed as box plots. **P < 0.01 (ANOVA and Tukey HDS test). I) A7 cells were transduced with lentiviral vectors encoding Flag-FilGAP (WT, 2A, KR/A, and KR/A-2A) and subjected to chemotactic invasion assay. J–K) The velocities and directionalities of migrating cells were calculated, and the data were expressed as box plots. **P < 0.01 (ANOVA and Tukey HDS test). Scale bar: 50 μm.

Finally, we examined the role of phosphorylation of FilGAP in chemotactic cancer cell invasion against EGF gradient. First, we tried to establish an experimental method for chemotaxis in a three-dimensional environment by modifying the methods of a previous publication (Fig. 7A, see Materials and Methods) (37). We have successfully generated linear EGF gradients in the three-dimensional collagen gels, and this slope was maintained for 20 h (Fig. 7B–D). A7 cells showed little or no movement in the absence of EGF, but in the presence of uniform EGF they showed high motility at random (Fig. 7E). Furthermore, under the EGF gradient, cells extended their protrusions in the direction of the EGF gradient and continued sustained movement in the direction (Fig. 7F). In EGF gradient, cell motility speed was increased compared to a uniform concentration of EGF and exhibited very high directionality (Fig. 7G and H, Movie S3). FilGAP WT and KR/A expressing cells show highly chemotactic properties against EGF-like nontransduced control cells, whereas FilGAP 2A and KRA-2A mutants expressing cells were not (Fig. 7I and Movie S4). Interestingly, both speed and directionality were reduced in FilGAP 2A mutant expressing cells, whereas directionality but not speed was reduced in FilGAP KRA-2A-expressing cells (Fig. 7J and K). FilGAP 2A-expressing cells appear to be unable to move as they continue to extend multiple protrusions in different directions. In contrast, KRA-2A-expressing cells frequently changed their direction by moving their protrusions in and out. Given that FilGAP 2A stabilized FA in the presence of EGF but KRA-2A did not (Fig. 6A–C), it is possible that stabilization of cell-extracellular matrix (ECM) adhesion reduces cell motility and suppression of lamellipodia is necessary for directional change.

**Fig. 8.**
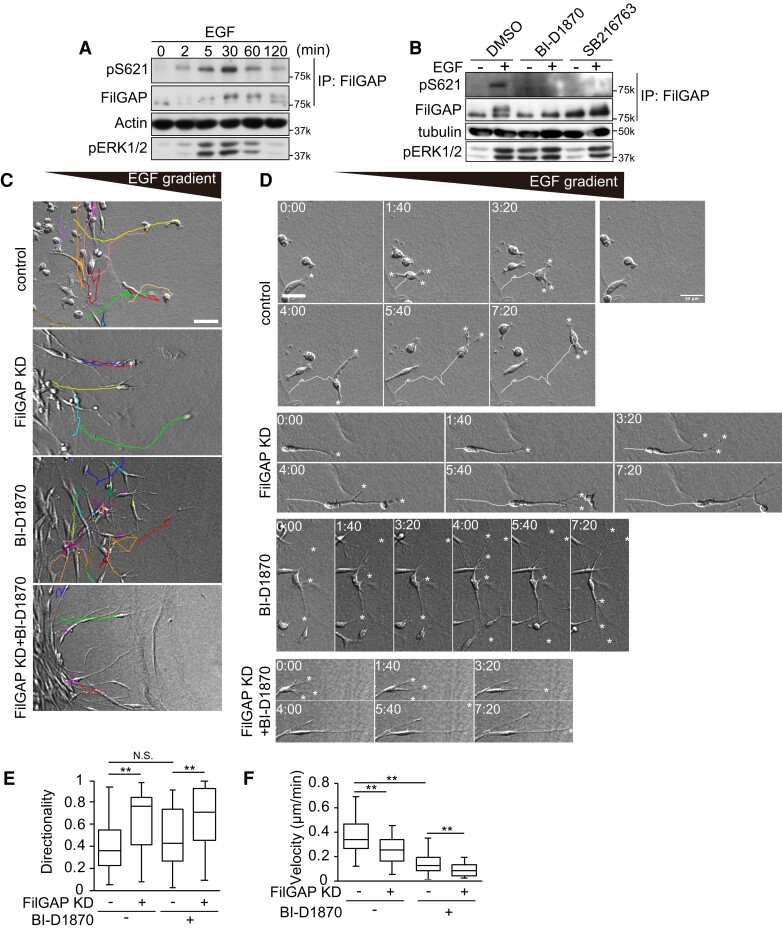
Depletion of FilGAP enhances directionality of cancer cell chemotactic invasion. A) Serum-starved MDA-MB-231 cells were treated with EGF for indicated time. Phosphorylation of FilGAP at Ser621 was examined by western blotting after immunoprecipitation. B) Serum-starved MDA-MB-231 cells were incubated in the presence of indicated inhibitors for 2 h and then treated with or without EGF for 30 min. Phosphorylation of FilGAP was evaluated by western blotting with anti-pSer621 FilGAP antibody. C) MDA-MB-231 cells were subjected to three-dimensional chemotaxis assay. Differential Interference Contrast (DIC) images and cell trajectories between 4- and 18-h during assays. D) Montage images every 100 min. Trajectories of migrating cells are indicated by white lines, and tips of protrusions are indicated by asterisks. E and F) The velocities and directionalities of migrating cells were calculated, and the data were expressed as box plots. **P < 0.01 (ANOVA and Tukey HDS test). Scale bars: 50 μm.

Because A7 cells express very low levels of FilGAP (26), MDA-MB-231 breast carcinoma cells were used to investigate the role of endogenous FilGAP in three-dimensional chemotaxis. Endogenous FilGAP was phosphorylated in response to EGF treatment in MDA-MB-231 cells and this was suppressed by RSK and GSK3 inhibitor treatments (Fig. 8A and B). In EGF gradient, MDA-MB-231 cells changed their direction of movement more frequently than A7 cells and showed lower directionality (Fig. 8C–E and Movie S5). When FilGAP expression was suppressed by siRNA (FilGAP KD), MDA-MB-231 cells extended their protrusions toward the direction of EGF and continued to migrate persistently toward that direction (Fig. 8C–E). In the presence of the RSK inhibitor, cells extended multiple protrusions into different directions, as in FilGAP 2A-expressing cells, and motility was greatly inhibited (Fig. 8D–F). However, even in the presence of RSK inhibitors, when FilGAP expression was suppressed, the cells continued to extend protrusions toward EGF, but the cells could hardly move from their position. These data suggest that FilGAP is crucial for protrusion formation downstream of RSK toward EGF in cancer cell chemotaxis, but that other pathways may play an important role in the regulation of motility.

## Discussion

**Fig. 9.**
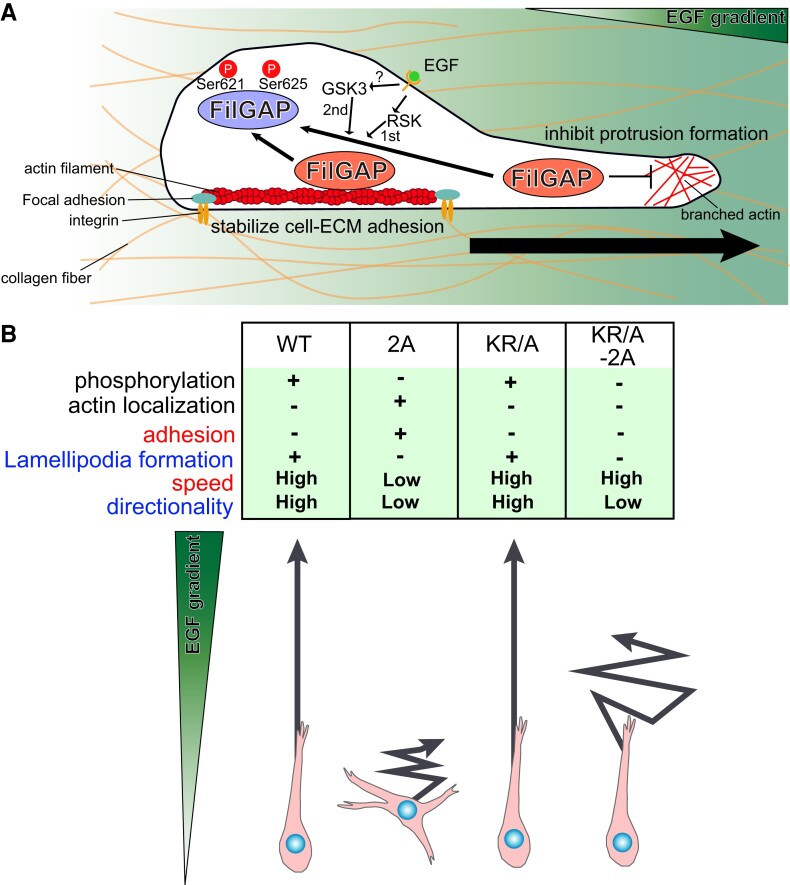
Summary of the effects of phosphorylation and localization of FilGAP on cell protrusions and focal adhesions and its possible relevance to chemotactic invasion. A) Schematic diagram of mechanistic of FilGAP function in cancer cell chemotaxis against EGF gradient. B) Comparison of the effects of FilGAP mutants on phosphorylation, localization to actin cytoskeleton, adhesion, lamellipodia formation, and chemotactic invasion in the presence of EGF.

In this study, we found that FilGAP is sequentially phosphorylated at Ser625 and Ser621 by RSK and GSK3 downstream of EGF signaling. Phosphorylation of FilGAP induced by EGF releases FilGAP from the actin cytoskeleton. We found that basic amino acid residues of FilGAP are critical for its localization to the actin cytoskeleton and successfully generated a mutant that could not localize to actin cytoskeleton. Phosphorylation of FilGAP induced by EGF reduced the FA area in the cells and increased the lamellipodia formation. Finally, we showed that nonphosphorylatable FilGAP mutant strongly suppresses the chemotactic cancer cell invasion toward EGF gradient. These data indicate that phosphorylation of FilGAP downstream of EGF is a regulatory pathway that promotes cancer chemotaxis (Fig. 9A).

We identified EGF-dependent phosphorylation sites of FilGAP as Ser625 and Ser621, and RSK and GSK3 as their responsible kinases by Phos-tag SDS–PAGE analysis (Figs. 1 and 2). Ser625 matched the phosphorylation consensus sequence of RSK (R/K-X-X-pS/T) (38). The phosphorylation of Ser625 then acted as a priming phosphorylation, and Ser621 was subsequently phosphorylated by GSK3, which is required for +4 position priming phosphorylation to recognize the substrate (18). It is generally accepted that GSK3 is phosphorylated at Ser9 by AKT downstream of growth factor signaling and inactivated at Ser 9 (18). The result that FilGAP is phosphorylated by GSK3 downstream of EGF seems to be inconsistent with previous reports. Recently, by phospho-quantitative analysis using Phos-tag SDS–PAGE, it has been shown that GSK3, which is phosphorylated and inactivated by insulin stimulation, is a fraction of the total GSK3 (39). In addition, there is a report that GSK3 is required for EGF-induced lamellipodia formation (40). Thus, FilGAP, whose Ser625 is phosphorylated by RSK downstream of EGF, is likely to be phosphorylated by a large fraction of GSK3, which is still active after EGF stimulation.

When FilGAP was phosphorylated at Ser621 and Ser625 downstream of EGF, it was released from the actin cytoskeleton (Fig. 3). However, actin cytoskeletal localization of FilGAP S621/625A is not different from FilGAP WT in the absence of EGF (Fig. 3B–D). Thus, the localization of FilGAP to actin cytoskeleton seems to be regulated by various signaling pathways. Indeed, we previously reported that FilGAP is phosphorylated at multiple serine/threonine residues including Ser402 in the middle of FilGAP sequence (391–452 aa), and this also releases FilGAP from the actin cytoskeleton (22, 29). FilGAP mutants in which these serine/threonine residues were replaced with alanine (ST/A mutants) markedly increased the localization of FilGAP to the actin cytoskeleton, even in the absence of serum or growth factors (Fig. 4F). In any case, downstream of EGF, phosphorylation of Ser621/Ser625 is important for the regulation of FilGAP localization to the actin cytoskeleton, which may be important for cancer cell chemotaxis.

We identified basic amino acid residues of FilGAP as a critical region for its localization to the actin cytoskeleton (Fig. 4). The molecular mechanism by which this FilGAP actin-localization motif is involved in the regulation of its cellular localization remains to be determined. The direct binding of FilGAP to F-actin was examined by a sedimentation assay using polymerized actin, but no clear binding was observed (data not shown). Localization of FilGAP to actin cytoskeleton may require interaction with other actin-binding proteins. We also showed that C-terminal deletion of FilGAP also affects its localization to Triton X-100 insoluble precipitate (Fig. 4B). The C-terminal region of FilGAP includes filamin A binding region and coiled-coil domain for dimerization (22, 41). In addition to the basic motif, binding to filamin A and dimerization may be involved in a complex manner in the regulation of localization to actin cytoskeleton.

FilGAP, which cannot localize to actin cytoskeleton, was also able to inhibit lamellipodia formation and phosphorylation of FilGAP at Ser621 and Ser625 attenuates this activity (Fig. 5). The lamellipodia formation requires the production of phosphatydil-inositol-3,4,5 triphosphate (PIP3) by activation of PI3K and the activation of Rac1 in the plasma membrane downstream of growth factors (42). FilGAP binds to PIP3 and Arf6 via its pleckstrin homology (PH) domain, and this binding is required for membrane targeting and inactivation of Rac1 (34, 43). We showed that FilGAP phosphorylated at Ser621 was decreased in the cell periphery (Fig. 3A). Phosphorylation may directly or indirectly affect the binding of FilGAP to PIP3 or Arf6 through PH domain.

We found that FilGAP, which can be associated with the actin cytoskeleton, increases FA (Fig. 6). FA, matured adhesion, is connected to actin stress fibers and induced by active RhoA (35). There have been several reports that Rac1 activation reduces FA (44, 45). Since Rac1 and RhoA antagonize each other (46), the absence of FilGAP on actin cytoskeleton may have inhibited FA formation by inducing Rac1 activation and RhoA inactivation. Assembly of FA is also induced by mechanical tension. Interaction between FilGAP with filamin A has been suggested to be involved in mechanotransduction (47–49). FilGAP localized on actin stress fibers may also be involved in the FA formation by generating tension in actin stress fibers via interaction with filamin A.

We established a three-dimensional chemotaxis assay (Fig. 7). In this assay, the cells extended their protrusions in the direction of higher concentrations of EGF and moved persistently in the same direction, confirming the validity of this method as a chemotaxis assay. We showed that phosphorylation of FilGAP is required for effective cancer cell chemotaxis in a three-dimensional environment (Fig. 7, Fig. 9A). A table comparing the phosphorylation and localization of various FilGAP mutants with their effects on chemotaxis is shown in Fig. 9B. From this table, it appears that FilGAP may regulate the direction of protrusion and the speed of cell motility through the inhibition of protrusion and adhesion, respectively. FilGAP 2A-expressing cells showed lower motility and directionality compared to FilGAP WT. On the other hand, FilGAP KR/A-2A-expressing cells showed high motility but frequently changed their direction. The exact reason for the difference in effects on cell motility between the two mutants is still unclear, but since FilGAP 2A stabilizes FA but KR/A-2A does not, we speculate that the difference may be due to the strength of the adhesion to the extracellular matrix that serves as the scaffold. FilGAP 2A-expressing cells extend their cell protrusions in multiple directions and appear to be unable to retract their protrusions, possibly because they are too strongly bound to collagen fibers. On the other hand, FilGAP KR/A-2A mutant strongly suppressed cell protrusion formation, so it is possible that the cells were unable to maintain their protrusion for a long time and frequently changed direction.

The molecular mechanism of chemotaxis has been elucidated by experiments using lymphocytes and Dictyostelium. During chemotaxis, local PIP3 production and RacGEF accumulation occur at the leading edge, followed by Rac activation, which generates actin polymerization and propulsion of the cell forward (50–52). The function of RacGAP in chemotaxis has not been well characterized compared to RacGEF. β-chimerin, a Rac-specific GAP, regulates T-cell adhesion and chemotaxis in a diacylglycerol-dependent manner (53). FilGAP inhibits cell polarization and motility evoked by Stromal cell-derived factor-1 in pro-B lymphocytes (54), suggesting that phosphorylation of FilGAP by RSK/GSK3 is also involved in lymphocyte chemotaxis.

In conclusion, we have shown the localization of FilGAP is regulated by phosphorylation downstream of RSK/GSK3 signaling in cancer cell chemotaxis, which regulates adhesions and protrusions. This is the first paper to reveal the detailed behavior of RacGAP in cancer cell chemotaxis, and it is hoped that future studies will elucidate its role in cancer dissemination.

## Materials and methods

### Plasmids and reagents

Flag-FilGAP in pCVM5 vector was constructed as described previously (22). Flag-FilGAP (1–391, 372–748, 552–748) was constructed as described previously (30). Primers used in this study were listed in Table S1. Flag-FilGAP (649–748, 578–748) was generated by PCR. Flag-FilGAP amino acids substituted mutants were generated by site-directed mutagenesis kit (Stratagene). EGFP-EV4 was generated by inserting a PCR-amplified 4xEV linker into Bgl II in pEGFP-C1. pEGFP-EV4-FilGAP was constructed by inserting the EcoRI/BamHI fragment of FilGAP into the EcoRI/BamHI site of pEGFP-EV4-C1. Lentiviral vectors CSII-EF-MCS, provided by H. Miyoshi (RIKEN). CSII-EF-MCS-HA-FilGAP and CSII-EF-MCS-IRES-puro were constructed as described previously (24, 27). Flag-FilGAP (WT, S621/625A, KR/A, KR/A-S621/625A) were cloned into CSII-EF-IRES-puro by PCR. Paxillin cDNA was amplified from HEK293T cells by RT-PCR and directedly cloned into CSII-EF-MCS-mCherry-IRES-puro vector by in-Fusion HD cloning kit (Takara, Japan). Lentiviruses were produced in HEK293T cells. Antibodies and reagents used in this study was listed in Table S2. Antiphosphorylated Ser621 FilGAP polyclonal antibody was generated by SCRUM (Tokyo, Japan) using phosphopeptide, SVGGRSpSRAT at human FilGAP protein residues 615-624. CA-MEK1 was provided by E. Nishida (RIKEN). pCMV5-GSK3β was provided by S. Hisanaga (Tokyo metropolitan University).

### Cell culture and transfection

HEK293T cells, MDA-MB-231 cells, and COS-7 cells were maintained in Dulbecco's modified Eagle's medium supplemented with 10% fetal calf serum, 50 units/mL penicillin, and 100 mg/mL streptomycin (Thermo Fisher Scientific). A7 cells were maintained in minimum essential medium eagle (Sigma) supplemented with 8% newborn calf serum, 2% fetal calf serum (Gibco), 50 units/mL penicillin, and 50 mg/mL streptomycin. For transfection, cells were transfected with plasmid DNA or siRNA using Lipofectamine 2000 or Lipofectamine RNAi max (Invitrogen) according to the manufacturer's instructions.

### Phosphorylation analysis

Phos-tag SDS–PAGE was performed with use of 10% (w/v) polyacrylamide gels containing 25 μM Phos-tag acrylamide (Wako chemicals) and 100 μM MnCl_2_. Cells were lysed in Radio-immunoprecipitation assay (RIPA) buffer without ethylenediaminetetraacetic acid (EDTA) (20 mM Tris–HCl (pH 7.5), 120 mM NaCl, 1% Triton X-100, 0.5% sodium deoxycholate, 0.1% sodium dodecyl sulfate, 10 mM MgCl_2_, 10 mM NaF, 10 mM β-glycerophosphate, protease inhibitor cocktail, and 1 mM dithiothreitol (DTT)). Cell lysates were centrifuged at 10,000 × *g* for 5 min, supernatant fluids were subjected to immunoblotting analysis. For in vitro kinase assay, Flag-FilGAP or Flag-GSK3β was expressed in HEK293T cells, immunoprecipitated by anti-Flag agarose, and eluted with Flag peptide. A7 cells were serum starved and stimulated with EGF for 30 min and then lysed in lysis buffer (20 mM Tris–HCl 7.5, 100 mM NaCl, 2 mM MgCl_2_, 1 mM ethylene glycol tetraacetic acid (EGTA), and 1% Triton X-100) and immunoprecipitated with anti-RSK antibody or normal mouse IgG. Anti-RSK immunoprecipitated was washed three times by lysis buffer and two times by Kinase buffer (20 mM Tris–HCl (pH7.5), 5 mM M MgCl_2_). Purified Flag-FilGAP was incubated with anti-RSK (or normal IgG) immunoprecipitates or purified Flag-GSK3b in the presence of 1 mM ATP for 30 min at 35 °C.

### Subcellular fractionation

A7 cells or human embryonic kidney 293 (HEK293) cells were lysed in Cytoskeletal (CSK) buffer (20 mM PIPES (pH 6.8), 2 mM MgCl_2_, 50 mM KCl, 5 mM EGTA, 1 mM DTT, and 0.5% Triton X-100), and cell lysates were centrifuged at 10,000 × *g* for 5 min. Supernatant was removed and the precipitate was sonicated after suspension in an equal volume of CSK buffer.

### Microscopic analysis

For immunofluorescence staining, cells were washed twice with phosphate-buffered saline (PBS) and fixed with 3.7% formaldehyde for 20 min. For cytoskeletal staining, cells were washed once, permeabilized in CSK buffer for 2 min, and then fixed in with 3.7% formaldehyde for 20 min. Fixed cells were permeabilized with 0.5% Triton X-100 in PBS for 5 min and immunostained with primary antibodies. Cells were then washed and incubated with Alexa-Fluor-dye-labeled secondary antibodies (Invitrogen). After washing with PBS, cells were observed under an Olympus IX81 fluorescence microscope (Olympus, Tokyo, Japan). Images were acquired by a charge-coupled device (CCD) camera (ORCA-ER; Hamamatsu Photonics, Hamamatsu, Japan) or an Electron multiplying CCD (EMCCD) camera (iXon3; Andor, South Windsor, CT, USA). Confocal images were obtained using Olympus DSU (Disk scanning unit)-IX81 with a 100× objective.

### Live cell imaging of focal adhesion

A7 cells were transduced with mCherry-Paxillin by lentivirus and selected with puromycin. mCherry-Paxillin-expressing A7 cells were transfected with EGFP-EV-FilGAP and cultured in collagen-coated glass bottom dishes (MatTek) in a medium containing 1% serum. Twenty-four hours after transfection, 50 ng/mL EGF was added and observed under IX83 fluorescence microscope (Olympus, Tokyo, Japan) equipped with Zyla 4.2-Plus USB 3.0 scientific complementary metal-oxide-semiconductor (sCMOS) camera (Andor) for 6 h at 2 min intervals. The lifetime, assembly rate, and disassembly rate of focal adhesions were measured according to a previous report (36). Briefly, region of interest (ROI) was drawn closely around a FA, as well as a noncell area for background control. The fluorescence intensity of an ROI was measured at all time points from the beginning of assembly to its disassembly, and the background signal was subtracted. The data was further processed with a running average of three frames and a lifetime curve was created. Curve fitting was performed using “Solver” add-in on Excel software.

### Three-dimensional chemotaxis invasion assay

Three-dimensional chemotactic invasion assay was performed according to the previous publication with some modification (37). Briefly, a 90 μL droplet of collagen I (rat tail acid extracted, BD Biosciences) at a final concentration of 1.7 mg/mL was put on the center of the glass bottom dish (MatTek) and incubated for 1 h at 37 °C. After collagen gel polymerization, a silicon tube was placed on the center of the gel, and a 30 μL droplet of collagen I at a final concentration of 2.5 mg/mL containing 5 μg/mL alexa568-EGF (Invitrogen) was put in the silicon tube. The cells suspension including 1.5 × 10^5^ cells was placed outside of the silicon tube and incubated for 1 h to allow the cells to adhere. After the medium and silicone tube were removed, 1.5 mL of 3 mg/mL atelocollagen solution was layered over the cells and incubated at 37 °C for 2 h. After the addition of 1 mL medium, cells in the outer gel were then imaged every 10 min for 24 h by difference interference contrast video microscopy using a dry objective at the interface of the inner and outer gels. Fifty to hundred cells were manually tracked and their velocity and directionality were analyzed with the Chemotaxis and Migration tool image J plugin (ibidi).

### Statistical analysis

Statistical analysis has been performed using one-way analysis of variance followed by an all pairwise multiple comparison procedure (Tukey HSD test). Data in Figs. 3D, E, G, I, and 5E, G have been tested using Student's t test.

## Supplementary Material

pgae071_Supplementary_Data

## Data Availability

All data are included in the manuscript and supplementary material.
