# Supplementary material for: RSK/GSK3–mediated phosphorylation of FilGAP regulates chemotactic cancer invasion
Source: PNAS Nexus. 2024 Feb 9;3(2):pgae071. doi: 10.1093/pnasnexus/pgae071 (PMC10904226; doi:10.1093/pnasnexus/pgae071)
Supplement: pgae071_Supplementary_Data [file pgae071_supplementary_data.zip › PNASNEXUS-PNASNEXUS-2023-00618-T-s07.docx]

Dec. 28, 2023

We thank to the reviewers of our manuscript we submitted to *PNAS* for their valuable and helpful comments. We have revised the manuscript and presented additional data according to the reviewers’ comments. We have highlighted in red the text of the revised manuscript. We hope that the revised manuscript is acceptable for publication in *PNAS Nexus*.

Thank you very much for your consideration.

Sincerely,

Koji Tsutsumi PhD,

Associate professor

Division of Cell Biology

Department of Biosciences

School of Science, Kitasato University

1-15-1 Kitasato, Sagamihara

Minami-ku, Kanagawa 252-0373, Japan

TEL: +81-42-778-9410 Fax: +81-42-778-9942

E-mail: [k.tutumi@kitasato-u.ac.jp](mailto:k.tutumi@kitasato-u.ac.jp)

Reviewer#1

*This manuscript addresses the phosphorylation and localization of FilGAP, a Rac-specific GTPase that inactivates Rac and suppresses lamellipodia formation. The study identifies a RSK phosphorylation site on FilGAP (Ser625) as a priming site and a GSK3 phosphorylation site (Ser621) as a 2nd site of phosphorylation. The biochemistry is sound and rigorous, including phosphosite mapping and the generation of a new phospho-specific antibodies. The study further identifies a KR basic region responsible for binding to actin and shows that that phosphorylation releases FilGAP from cytoskeleton. The phospho-species promotes cell migration, as the phospho-dead mutant reduced migration speed and persistence towards an EGF gradient. However, the exact role of cytoskeleton versus cytoplasmic FilGAP in cell migration are less clear.*

We would like to thank Reviewer #1 for valuable suggestions and helpful comments. In this revised manuscript, we included new data and have reorganized the text in accordance with the reviewers’ comments.

*The authors conclude that RSK/GSK3 phosphorylation of FilGap inhibits the lamellipodia suppression activity of FilGap, so that phosphorylated species allows more protrusion and the phospho-dead 2A mutant has less protrusions. This activity may be a result of release from the cytoskeleton, as the KR mutant species that does not bind to the cytoskeleton also has more protrusion.*

Protrusion formation was strongly inhibited in the non-phosphorylated KRA mutant (KRA-2A), suggesting that the inhibition of protrusion formation by FilGAP is not due to its release from the cytoskeleton by phosphorylation. Our results suggest that at steady state there are two populations of FilGAP, one bound to the cytoskeleton and one unbound. When cells are stimulated by EGF, both of them are phosphorylated, and the one localized in the cytoskeleton is released from the cytoskeleton, and the FilGAP of either population is phosphorylated and hence cannot inhibit the protrusion formation.

*-Fig. 5B, C: The authors state that the 2A mutant has less protrusion early in a spreading assay, but more at the end. A discussion of the significance of the 2 phases of spreading would help with interpretation. Movie S1 does not play so cannot be interpreted. The plot in Fig. 5G calculates the number of cell with protrusions and shows 2A has less protrusions. However, a calculation of number of cells with >1 protrusion would be more useful and frequency or protrusion direction changes is needed to put the later data in context with FilGAP's overall regulation of protrusion and cell migration.*

We would like to thank the reviewer for valuable comments. We added a discussion of the significance of the two phases of spreading according to the reviewer's comments. We apologize for any inconvenience that MovieS1 cannot be played. We have replaced it with another file.　We measured the proportion of cells with >1 actin-rich protrusion formed by EGF treatment, and the results are shown in Figures 5E and 5G.

*The authors also show that the unphosphorylated cytoskeleton-localized FilGAP increases FA area, while the phospho-species and cytoplasm-localized KR mutants have less FA area. The authors conclude that the unphosphorylated FilGAP 2A stabilizes focal adhesions. 
- focal adhesion assembly rate, stabilization duration, and disassembly rates should be measured. In addition, the number and size of individual adhesions, at the protruding versus trailing edge were not measured. Without these specifics, putting the focal adhesion regulation in context with the findings on cell migration, chemotaxis, and invasion is very difficult.*

To investigate the mechanism by which FilGAP regulates focal adhesion, we generated cells stably expressing mCherry-Paxillin and examined the effect of FilGAP WT or 2A expression on the dynamics of focal adhesion. Although FilGAP did not significantly affect the assembly and disassembly rates, FilGAP 2A significantly increased the lifetime of focal adhesion (Figures 6G-K).

*-The summary model figure 8 does not help inform the literature. The conclusion that "phosphorylation of FilGAP downstream of EGF-signaling plays a critical role in regulating chemotactic tumor cell migration by controlling cell-matrix adhesion and protrusion formation" is sound. However, other ideas put forth, that attempt to explain more of the mechanism of FilGap action, require more study. In particular, "suppression of lamellipodia is necessary for directional change" and " it is likely that the cells were unable to maintain their protrusion for a long time and frequently changed direction" are very interesting and warrant study. However, current data do not well substantiate these claims. It should be worded that, at this time, these are hypothesis that explain the data. Without the mechanistic insight of which adhesions are controlled and how frequently protrusion direction changes occur, the paper is somewhat limited in scopt.*

As the reviewer pointed out, the data presented here on the regulation of protrusion and focal adhesion by FilGAP is not sufficient to explain the results of cell motility. We have revised the text to soften the statement and present our thoughts as speculation.

Rviewer#2

*A manuscript titled "RSK/GSK3 mediated phosphorylation of FilGAP regulates chemotactic cancer invasion" was examined for potential publication in PNAS. In this study, the authors show that FilGAP, a Rac-specific GAP, is phosphorylated in response to EGF treatment. Several phosphorylation sites are identified, with one site being apparently dependent on the other. The authors show that Ser625 is phosphorylated by RSK, which primes the phosphorylation of Ser621 by GSK3. Using unphosphorylatable mutants, the authors mainly show that phosphorylation of FilGAP induces its dissociation from actin filaments. They also show that the expression of a non-phosphorylatable FilGAP mutant reduces cell migration speed and persistence towards a gradient of EGF. 

Overall, the study reports interesting findings on new phosphorylation events associated with FilGAP downstream of the Ras/MAPK pathway. They report the interesting possibility that RSK primes FilGAP for phosphorylation by GSK3, and that unphosphorylatable mutations in FilGAP negatively affects its function in cell migration. The paper is also relatively well written, and the data are well presented. However, there are several issues in the interpretation of the data and their conclusions. There is also a disconnect between the first part of the paper on the characterization of FilGAP phosphorylation, with the analysis of phosphorylation site mutants using functional assays. While RSK and GSK3 may indeed regulate FilGAP phosphorylation, it would have been interesting to determine their role in FilGAP activity and not only rely on phosphorylation site mutants. Please find below several issues that would need to be addressed:*

We would like to thank Reviewer #2 for valuable suggestions and helpful comments. In this revised manuscript, we included new data and have reorganized the text in accordance with the reviewers’ comments. We have added the results to show the effect of FilGAP phosphorylation on localization, protrusion, and focal adhesion using inhibitors of upstream kinases (Figure 3, 5 and 6). We have shown that FilGAP Ser621 is directly phosphorylated by RSK/GSK3 in vitro using purified proteins (Fig.2D).

*Main concerns:*
*1) In figure 1, the rationale leading to the selection of phosphorylation sites is confusing. On line 116, the authors should at the very least specify what are the 6 previously identified phosphorylation sites, and what kinase(s) might regulate them.*

We have added the sentence to explain the detailed information about phosphorylation sites we previously reported.

*2) Also in figure 1, the authors should include loading controls whenever phostag blots are shown (tubulin). The levels of expression constructs should be included without the addition of phostag through a simple Flag blot, particularly since figure 2A shows that a shift is visible without phostag. As well, the phospho-ERK status should be included to verify EGF stimulations, including total ERK levels.*

Anti-Flag blot on SDS-PAGE without the addition of phostag (Laemmli) was added to Fig. 1A to show that the expression levels of FilGAP are the same. We also added blots for tubulin, pERK1/2, and total ERK1/2. The results show that without the addition of phostag, the mobility of FilGAP is almost the same with and without EGF treatment and the same in WT and ST/A mutants.

*3) Line 120: I don't understand the logic here, as it is unclear why the authors have excluded the region between aa 391 and 577. Clearly, the 373-748 mutant appears to shift, and the corresponding sites may not all be within the 552-748 mutant. In other words, the authors should test a mutant between 391 and 577 to verify whether this region contains EGF-responsive phosphorylation sites.*

FilGAP 552-748 aa and 577-748 aa were phosphorylated but 649-748 aa was not, indicating that 577-648 aa contains phosphorylation sites and we showed Ser621 and Ser625 in this region are actually phosphorylated. However, as reviewer #2 pointed out, it is possible that phosphorylation sites may also be present in 373-552aa. We have changed the wording.

*4) Line 128: The results suggest that phosphorylation of S625 is required for S621 phosphorylation but does not prove it. The conclusion should be softened to reflect this.*

We thank the reviewer for the kind suggestion. As reviewer #2 pointed out, we changed the wording of the sentence.

*5) In the first section of the Results section, the authors should inform whether any of the identified sites have previously been identified, such as using information from Phosphositeplus. Also, potential kinases can be predicted using the Kinase Library, which is also available through Phosphositeplus.* 

We thank the reviewer for the kind suggestion. We searched the phosphorylation sites of FilGAP using Phosphositeplus and found phosphorylation of Ser625 is reported in phospho-proteome analysis. We added the sentences to the manuscript explaining this.

*6) The phospho-S621 antibody appears to be nicely specific. Does it work against endogenous FilGAP? What about endogenous protein regulation by RSK and GSK3?*

Sensitivity of anti-phospho-FilGAP Ser621 are not so good. This antibody can detect phosphorylation of overexpressed FilGAP but not endogenous FilGAP in cell lysates. However, this antibody can detect phosphorylation of endogenous FilGAP if after concentrated by immunoprecipitation with FilGAP antibody. We have shown the phosphorylation Ser621 of endogenous FilGAP in MDA-MB-231 cells in Figure 8A and B after immunoprecipitation. Both RSK and GSK3 inhibitors suppressed phospho-FilGAP Ser621 induced by EGF treatment.

*7) In figure 2B, it is unclear why the authors have not used their phospho-S621 antibody, as shown in figure 2A. Also, why not use full-length FilGAP (as in 2A)?*

We thank the reviewer for the kind suggestion. We added the blot of phospho-S621 to Figure 2B. We used 577-748 because it is difficult to distinct phospho-Ser625 and phospho-Ser621 using full length FilGAP. We added the sentence explaining why we used FilGAP 577-748.

8) *Line 143: The authors should be careful in the way they summarize results. Instead of saying "FilGAP-Ser621 was not phosphorylated by GSK3 overexpression.", they should say that GSK3 overexpression did not promote FilGAP-Ser621 phosphorylation. The meaning is different and the latter more accurate. Same for the next sentence, as it should say that CA-MEK1 overexpression promotes Ser621 phosphorylation. In addition, it would be useful to add a substrate of GSK3 to show that its overexpression can indeed promote the phosphorylation of some substrates under these conditions.*

We have corrected the manuscript in accordance with this comment.

*9) Line 146, the authors likely mean minus 2 rather than plus 2 position.*

As the reviewer #2 has pointed out, we have revised the text.

*10) A critical experiment would be to convert Ser625 into D/E to demonstrate that a negative charge indeed allows for GSK3 to phosphorylate Ser621. In vitro kinase activity assays are also missing in the study. These phosphorylation site mutants could also be used in IF experiments to determine actin colocalization.*

We tested whether the S625D mutant could be phosphorylated by GSK3 in the absence or presence of CA-MEK, but it was not. We suspect that this is because phosphomimetic mutants sometimes behave differently than when a phosphate group is actually added to the amino acid side chain. We performed *in vitro* reconstitution experiments using purified protein.　S621 was phosphorylated only in the presence of both RSK and GSK3, indicating that both RSK and GSK3 are required for the phosphorylation of S621. Together with the results of Fig. 1E and Fig. 2B, it is strongly suggested that FilGAP is phosphorylated downstream of EGF by RSK at S625 and then by GSK3 at S621.

*11) The fractionation results shown in figure 3 are not as clear as the authors conclude. First, the authors state that "The amount of FilGAP WT, but not S621/625A, in TritonX-100 insoluble precipitate was significantly decreased after EGF treatment (Fig. 3C, D)." This is not particularly evident, as FilGAP from cells treated with EGF is shifted and it is more difficult to determine total amounts. Second, the authors state that "... FilGAP phosphorylated at Ser621 was mostly collected in the soluble fraction ...". Here again, the result is not clear as there is not total FilGAP in the soluble fraction in the first place. Third, the total amount of FilGAP in panel F appears to change between conditions, making it very difficult to interpret the data. Fourth, the authors are not making use of inhibitors or agonists that lead to FilGAP phosphorylation, and thus could change its localization. Overall, this reviewer is not convinced about the S621-dependent changes in FilGAP subcellular localization.*

As the reviewer pointed out, FilGAP is phosphorylated and shifted upward by EGF stimulation, so it may be difficult to determine the amount of FilGAP from the appearance of the bands. Since EGF treatment decreases FilGAP in the precipitate while increasing FilGAP in the supernatant, the data are presented as a ratio of supernatant and precipitate. The effects of RSK or GSK3 inhibition on the localization of FilGAP were also examined (Fig. 3F, G).

*12) Please change the "CSK" term for Triton X-100 treatment as it is confusing and could mean other things.*

We changed “CSK” to “Triton-insoluble”.

*13) Line 152: typo.*

We found some typos and corrected them.

*14) The authors should explain why they have chosen A7 cells for their studies.*

We added the sentence why we use A7 cells.

*15) A tubulin blot is missing in panel 4F*

In this experimental group (n=3), we used actin and vimentin as loading controls. Since all the samples have been discarded, the experiment needs to be done again to add tubulin data. Under the conditions of this experiment, F-actin is recovered in the precipitates and G-actin is recovered in the supernatant. Therefore, we believe that actin can also be used as a loading control to confirm that the fractionation is successful. So, we have stated in the text that we used actin and vimentin as loading controls.

*16) In figure 5, the letters associated with panels A and B are missing. Also, the resolution of panel B is insufficient to conclude on the impact of the mutations on cell spreading. In addition, the control untreated condition is missing from panels B and C.*

We thank the reviewer for the kind suggestion. We added the letters A and B to the panels A and B. We changed the cell spreading photo to a higher resolution one.

*17) In figure 5, the authors have not taken advantage of agonists and inhibitors of the Ras/MAPK pathway and RSK, which would have consolidated their results. The expression of mutants and their associated changes in focal adhesions give limited insights into the role of FilGAP and its regulation.*

We examined the effect of RSK or GSK3 inhibitors on the protrusion formation and foal adhesion area (Fig. 5F, G, Fig. 6D-F). RSK inhibition promoted inhibition of protrusion formation by FilGAP WT and K/RA, supporting the data using mutants. While RSK inhibition increased focal adhesion not only in WT but also in KR/A expressing cells. RSK is known to inhibit integrin activation and inhibit focal adhesion formation through the phosphorylation of filamin. Therefore, we interpreted this result as being due to stabilization of focal adhesion downstream of RSK through pathways other than FilGAP.
