# Supplementary material for: RSK/GSK3–mediated phosphorylation of FilGAP regulates chemotactic cancer invasion
Source: PNAS Nexus. 2024 Feb 9;3(2):pgae071. doi: 10.1093/pnasnexus/pgae071 (PMC10904226; doi:10.1093/pnasnexus/pgae071)
Supplement: pgae071_Supplementary_Data [file pgae071_supplementary_data.zip › PNASNEXUS-PNASNEXUS-2023-00618-T-s01.docx]

**Supporting Information for**

RSK/GSK3 mediated phosphorylation of FilGAP regulates chemotactic cancer invasion

Koji Tsutsumi and Yasutaka Ohta

* Koji Tsutsumi

Email: [k.tutumi@kitasato-u.ac.jp](mailto:k.tutumi@kitasato-u.ac.jp)

**This PDF file includes:**

Figures S1 to S3

Tables S1 to S2

Legends for Movies S1 to S5

**Other supporting materials for this manuscript include the following:**

Movies S1 to S5


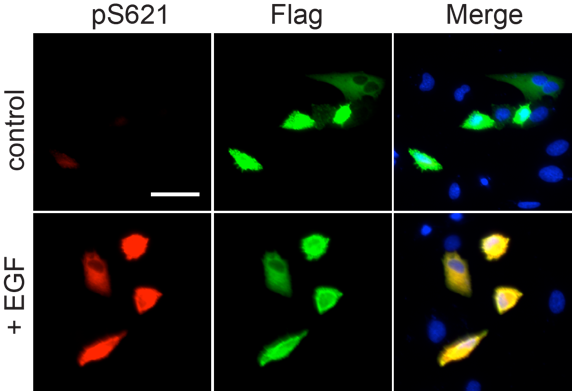


Fig. S1. pS621 is increased after EGF treatment. A7 cells were transfected with Flag-FilGAP and incubated with EGF for 30 min, and then fixed and stained with anti-pSer621 and anti-HA antibodies. Scale bars: 50 μm.


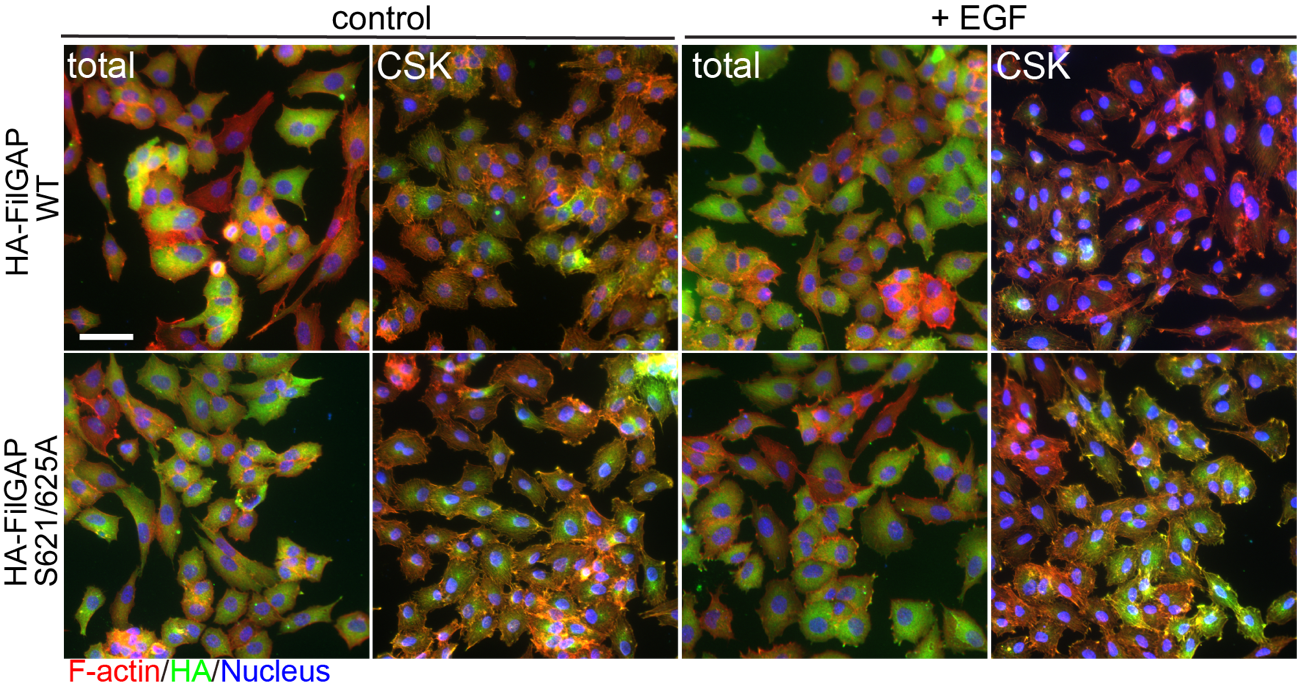


Fig. S2. Actin cytoskeletal localization of FilGAP is decreased after EGF treatment. HA-FilGAP (WT or S621/625A) transduced A7 cells were incubated in the presence or absence of EGF for 30 min. Cells were fixed after treatment with (CSK) or without (total) 0.5% Triton X-100 and stained with anti-HA antibodies for FilGAP and Alexa568 conjugated phalloidin for F-actin. Scale bars: 50 μm.

**
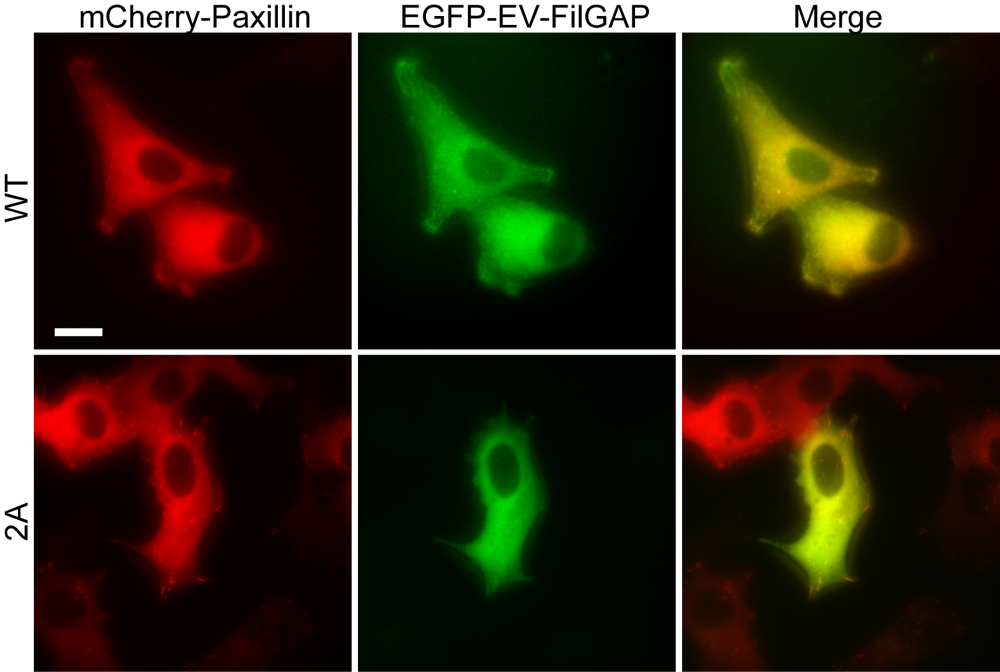
**

**Fig. S3. Expression of EGFP-EV-FilGAP.** mCherry-Paxillin transduced A7 cells were transfected with EGFP-EV-FilGAP (WT or 2A) and cultured O/N on collagen coated glass bottom dish in the reduced serum (1%). Scale bar: 20 μm.

Table S1. List of antibodies and regents used in this study

Table S2. Sequence of primers and siRNA used in this study

Movie S1. Cell spreading of Flag-FilGAP stably expressing A7 cells. Images were taken every 30 seconds and processed using Image J.

**Movie S2.** Time lapse images of mCherry-Paxillin transduced A7 cells. Images were taken every 2 minuts and processed using Image J.

Movie S3. Three dimensional chemotaxis assay of control A7 cells. Images were taken every 10 minutes and processed using Image J.

Movie S4. Three dimensional chemotaxis assay of Flag-FilGAP (WT, 2A, KR/A and KR/A-2A) expressing A7 cells. Images were taken every 10 minutes and processed using Image J.

Movie S5. Three dimensional chemotaxis assay of MDA-MB-231 cells. Images were taken every 10 minutes and processed using Image J.
